# *Dendrochilum hampelii* (Coelogyninae, Epidendroideae, Orchidaceae) traded as ‘Big Pink’ is a new species, not a hybrid: evidence from nrITS, *mat*K and *ycf*1 sequence data

**DOI:** 10.3897/phytokeys.56.5432

**Published:** 2015-10-01

**Authors:** Bobby P. Sulistyo, Ronny Boos, James E. Cootes, Barbara Gravendeel

**Affiliations:** 1 Naturalis Biodiversity Center, Leiden University, 2300 RA Leiden, The Netherlands; 2 University of Applied Sciences Arnhem and Nijmegen, 6525 EN Nijmegen, The Netherlands; 3 Centre for Australian National Biodiversity Research, Canberra, Australia; 4 University of Applied Sciences Leiden, 2333 CK Leiden, The Netherlands; 5 Institute Biology Leiden, Leiden University, 2300 RA Leiden, The Netherlands

**Keywords:** *Dendrochilum hampelii*, Molecular Phylogenetics, Orchids, the Philippines

## Abstract

In 2013, an unidentified species of *Dendrochilum* appeared in cultivation under the commercial trade name ‘Big Pink’. Using sequences of the nuclear ribosomal ITS1-5.8S-ITS2 region and of the plastid *mat*K and *ycf*1 genes, we examined the phylogenetic relationships between ‘Big Pink’ and six other species of the phenetically defined Dendrochilum subgen. Platyclinis sect. Eurybrachium. Separate and combined analyses (using Bayesian, Maximum Likelihood and Parsimony inference) showed consistent placement of the unidentified species within a statistically well supported clade. Furthermore, the multi-copy nrITS marker showed clear distinct peaks. Thus, we found no evidence that ‘Big Pink’ could be a hybrid. Against this background, and further supported by species-specific mutations in (at least) nrITS and *ycf*1, we formally describe ‘Big Pink’ as a new species under the name *Dendrochilum hampelii*. Morphologically, it is most similar to *Dendrochilum propinquum*, but it differs in a number of characters. Of the two cultivated individuals available for our study, one was of unrecorded provenance. The other allegedly originated from the Philippines. Observations of the species occurring in the wild in the Philippines in the northern provinces of Bukidnon and Misamis Oriental on the island of Mindanao confirmed this.

## Introduction

The largely Malesian genus *Dendrochilum* Blume (Blume 1825–1827: 398–399; published 1825) accommodates ca. 275 species—most of them described based on field inventories in the periods ca. 1900–1940 and ca. 1985–2000 (Pedersen 2007a). However, since the turn of the millenium, most new species of *Dendrochilum* have been described based on cultivated material of unrecorded provenance (cf. Pedersen 2011). Following the publication of protologues based on cultivated material, four of these species have been located in the wild (Cootes 2011). This is the case for *Dendrochilum coccineum* H.A.Pedersen & Gravend. (Pedersen et al. 2004), *Dendrochilum croceum* H.A.Pedersen (Pedersen 2005), *Dendrochilum quinquecallosum* H.A.Pedersen (Pedersen 2007b) and *Dendrochilum undulatum* H.A.Pedersen (Pedersen 2007b). The discovery of *Dendrochilum coccineum* in the Philippines confirmed a phylogeny-based hypothesis put forward by Pedersen et al. (2004). Similar phylogenetic inference of *Dendrochilum warrenii* H.A.Pedersen & Gravend. (Pedersen et al. 2004) as probably originating from the Philippines and/or Sulawesi (Pedersen et al. 2004) is still awaiting confirmation.

The formal description of new species of unknown natural distribution has undoubtedly served to stimulate the (partly successful) search for these species in the wild, thus demonstrating the relevancy of this practice—not least in a conservation context. Nevertheless, describing new species based on material in commercial trade also involves a few problems. Thus, Vermeulen et al. (2014) raised some issues of moral concern, and Pedersen (2011) emphasized that care should be taken not to accidentally describe artificial hybrids as new species. Until recently, this risk was negligible in connection with *Dendrochilum* since not a single *Dendrochilum* hybrid had been registered (cf. Pedersen 2011). However, now there are two *Dendrochilum* artificial hybrids registered in *The International Orchid Register* (http://www.apps.rhs.org.uk/horticulturaldatabase/orchidregister/orchidregister.asp; accessed on 13 February 2015), originating from Bornean parental species. This demonstrates that human assisted hybridization between different species of *Dendrochilum* is indeed possible—for which reason we must consider the possibility that seemingly undescribed species suddenly appearing in cultivation could in reality be artificial hybrids.

Since chloroplast DNA is usually maternally inherited and nuclear DNA is biparentally inherited in orchids, incongruences between nuclear and plastid gene trees might indicate past events of hybridization. For example, by comparing phylogenies based on cpDNA and nrDNA, Barkman and Simpson (2002) inferred a natural hybrid origin of *Dendrochilum acuiferum* Carr whereas Gravendeel et al. (2004) detected natural hybrids within the more distantly related genus *Pleione* D.Don. Further evidence for hybridization could potentially come from significant within-individual variation in multi-copy DNA markers.

This paper reports our study of an unidentified *Dendrochilum* (trade name: ‘Big Pink’) that appeared in cultivation in 2013. A live plant presented to the Hortus botanicus in Leiden carried a tag indicating a Philippinese provenance. However, as the plant came from a commercial nursery that trades much material of unknown geographic origin, we felt this provenance was in need of verification.

Synanthous inflorescences in combination with an entire rostellum, presence of stelidia and an apical wing on the column place the study plant in the phenetically defined subgenus *Platyclinis* Engl. as circumscribed by Pedersen et al. (1997). Within this subgenus, a firmly attached entire labellum and a stout and straight column with stelidia but without a foot place the plant in the phenetically defined section *Eurybrachium* Carr ex J.J.Wood, H.A.Pedersen & J.B.Comber (Pedersen et al. 1997). However, the morphology of ‘Big Pink’ does not match any previously described species in section *Eurybrachium*—implying that it should be formally described as a new species, provided it is not either an artificial or natural hybrid.

Altogether, we decided to examine ‘Big Pink’ in a molecular phylogenetic framework—and to describe it as a new species, if the results of the phylogenetic study could reject the possibility of ‘Big Pink’ being a hybrid.

## Methods

### Plant sampling and DNA extraction

The possible hybrid status of ‘Big Pink’ was tested using a molecular phylogenetic approach based on three markers, namely the biparentally inherited multi-copy nuclear ribosomal internal transcribed spacer (nrITS), and the maternally inherited plastid *mat*K and *ycf*1 genes. The ingroup consisted of ‘Big Pink’ and six other species belonging to Dendrochilum subgen. Platyclinis sect. Eurybrachium (cf. Pedersen et al. 1997), see Table 1.

**Table 1.** List of species sampled for our DNA-based phylogenetic analysis with voucher data. All species in the table belong to the phenetically defined Dendrochilum subgen. Platyclinis sect. Eurybrachium. Abbrevations of herbaria: C=University of Copenhagen, Copenhagen, Denmark; K=Royal Botanic Gardens Kew, United Kingdom; L=Naturalis Biodiversity Center, Leiden, The Netherlands; N=Nanjing University, Nanjing, China.

Species Voucher	NCBI GenBank accession numbers		
	nrITS	*mat*K	*ycf*1
*Dendrochilum apoense* T.Hashim. *cult. Hort. Bot. Hafn. s.n.* (C!)	KT334200	KT334206	KT334213
*Dendrochilum auriculare* Ames *cult. Hort. Bot. Hafn. P2012.5172* (C!)	KT334201	KT334207	KT334214
*Dendrochilum coccineum* H.A.Pedersen & Gravend. *cult. Richard C. Warren, Warren EQ 3060* (C!)	AY534923	KT334208	KT334215
*Dendrochilum convallariiforme* Schauer *cult. Hort. Bot. Hafn. P2012.5177* (C!)	KT334202	KT334209	KT334216
*Dendrochilum hampelii Sulistyo et al. *cult. Hort. bot. Leiden 20130654* (L! [WAG0116920]])	KT334203	KT334210	KT334217
*Dendrochilum septemnervium* H.A.Pedersen *cult. Hort. Bot. Hafn. P2012.5195* (C!)	KT334204	KT334211	KT334218
*Dendrochilum tortile* H.A.Pedersen *cult. Hort. Bot. Hafn. P2012.5200* (C!)	KT334205	KT334212	KT334219
*Thunia alba* (Lindl.) Rchb.f. Nepal, *Chase 589* (K!)	AY008466	AY121731	-
*Thunia alba* (Lindl.) Rchb.f. China, *B. Hou EThuA* (N!)	-	-	KF361675

* Trade name: ‘Big Pink’

A live plant of ‘Big Pink’ was available from the Hortus botanicus in Leiden, whereas the remaining *Dendrochilum* plants sampled for this study were reared in the Botanical Garden, Natural History Museum of Denmark. For information on vouchers, see Table 1. *Thunia alba* Rchb.f. was chosen as outgroup, based on the placement of the genus *Thunia* as sister to *Dendrochilum* using sequences from nrITS, *mat*K, *trn*L-F, and *rbc*L (Goldman et al. 2001, Gravendeel et al. 2001, van den Berg et al. 2005).

Total genomic DNA was obtained from 50 mg of silica dried or fresh leaf tissue. In the case of ‘Big Pink’, the tissue was mechanically reduced to dry powder using liquid nitrogen; for all other taxa, it was ground in Lysing Matrix A tubes (MP Biomedicals) and extracted using the DNeasy Plant Mini Kit (Qiagen) following the manufacturer’s protocol.

### Amplification and Sanger sequencing

The ITS1-5.8S-ITS2 region of the nuclear ribosomal internal transcribed spacer (nrITS) was amplified using primers 17SE (ACGAATTCATGGTCCGGTGAAGTGTTC) and 26SE (TAGAATTCCCCGGTTCGCTCGCCGTTAC), as described by Sun et al. (1994). Subsequently, a M13 universal sequencing primer was added to the 5’ end of the forward (TGTAAAACGACGGCCAGT) and reverse (CAGGAAACAGCTATGAC) primers to improve Sanger sequencing efficiency. Each PCR reaction consisted of 25 µl, containing the template DNA, CoralLoad PCR buffer (Qiagen), dNTPs, Taq DNA Polymerase (Qiagen), and both primers. The PCR reactions were carried out using a MyCycler Thermal Cycler (Bio-Rad) or a C1000 Touch Thermal Cycler (Bio-Rad). The thermal cycling protocol began with 5 min initial denaturation at 95 °C followed by 35 amplification cycles, each with 30 sec denaturation at 95 °C, 30 sec annealing at 50 °C, and 1 min extension at 72 °C, which were concluded by a 7 min final extension at 72 °C.

The primers for the amplification of the chloroplast *mat*K region were also used by Gravendeel et al. (2001), and specified as follows: 19F (CGTTCTGACCATATTGCACTATG) and 881R (TMTTCATCAGAATAAGAGT), 731F (TCTGGAGTCTTTCTTGAGCGA) and 2R (AACTAGTCGGATGGAGTAG). The PCR mix for the amplification of *mat*K followed that of nrITS, but with additional BSA. The thermal cycling protocol for *mat*K PCR began with 5 min initial denaturation at 94 °C followed by 28 amplification cycles, each with 30 sec denaturation at 94 °C, 30 sec annealing at 49 °C, 1 min extension at 72 °C, and ended with a 7 min final extension at 72 °C.

The 3’ end portion of the chloroplast *ycf*1 region was amplified using primers newly designed in this study. The design was based on the *ycf*1 sequences data set of Neubig et al. (2008) available on NCBI GenBank (http://www.ncbi.nlm.nih.gov/GenBank), specifically from the species of subfamily Epidendroideae. The sequences were aligned using Geneious 5.6.7 (Kearse et al. 2012), and conserved regions were identified to be used as annealing sites. The *ycf*1 sequences produced in this study had a complete marker size of approximately 1.5 kb. The *ycf*1 region was amplified using a Hot start PCR protocol with primers d147F (TGCAGCRAATTYATTTATGAGTC) and intR2 (GATTTGATTGGGATGATCCAAGG), d557F (TCAAGAGATCAAACCATKCAATCA) and 1560R (CTCTACGACGTCTGGGAGATAG). Each PCR reaction consisted of 25 µl, containing DNA template, Phire Hot Start II DNA Polymerase and Phire buffer (ThermoScientific), dNTPs, BSA, and both primers. The cycling condition started with 30 sec initial denaturation at 98 °C and followed by 30 cycles of 5 sec denaturation at 98 °C, 5 sec annealing at 65.5 °C, and 10 sec extension at 72 °C, concluded with a 1 min final extension at 72 °C.

Sanger sequencing of the amplification products was performed at Baseclear (http://www.baseclear.com/), using an ABI 3730xl sequencer (Applied Biosystems). All new sequences are deposited in NCBI GenBank (Table 1). All sequences of *Thunia alba* were obtained from NCBI GenBank.

### Phylogenetic analyses

Raw Sanger sequencing results in the form of AB1 files were edited and contigged using Sequencher 5.3 sequence analysis software (http://www.genecodes.com). The ends of all data sets were trimmed to avoid character misinterpretation. Ambiguous bases were replaced with “N” in the data matrix. The sequences were aligned using Geneious multiple sequence alignment in Geneious 5.6.7 (Kearse et al. 2012) with subsequent manual adjustments. Missing data were replaced with “?”.

Phylogenetic analyses were carried out by means of Maximum Parsimony and Maximum Likelihood using PAUP* and Bayesian methods using the software Bayesian Evolutionary Analysis and Sampling of Trees (BEAST ver. 1.8.0; Drummond et al. 2012). Both PAUP* and the BEAST program were used to analyze the nrITS, the combined plastid *mat*K + *ycf*1, and the combined nrITS + *mat*K + *ycf*1 data matrices. For the Bayesian analysis, the substitution and clock model was set as unlinked, and the chosen nucleotide substitution model was General Time Reversible (GTR), plus Gamma with 10 categories. The best fit substitution model for each partition was determined using the Find Model web tool (http://www.hiv.lanl.gov/content/sequence/findmodel/findmodel.html). A lognormal relaxed clock model was used for each partition, and the chosen tree prior was the Yule speciation process (Gernhard 2008). Tree samplings were generated through Markov Chain Monte Carlo (MCMC), with the number of generations set to 20,000,000 and a tree sampling for every 1000 generations. Three consecutive replicates were done to assess the consistency of the method. The three consistent replicates were then combined into a single matrix using LogCombiner c1.8.0 (http://beast.bio.ed.ac.uk/logcombiner) and used to search for the best probable tree using the program TreeAnnotator ver. 1.8.0 (http://beast.bio.ed.ac.uk/TreeAnnotator) with a 20% burnin value to avoid the reduction of posterior probability (PP) values, and visualized using FigTree ver. 1.4.2 (http://tree.bio.ed.ac.uk/software/figtree/).

## Results

The nrITS sequence alignment contained 854 positions with a mean ungapped length of 834 bp. Included in the alignment were the nrITS1 (236 positions), 5.8S RNA (166 positions), and mrITS2 (253 positions) regions. In total, the number of variable sites for the included positions was 164 (19.2%), of which 39 were potentially phylogenetically informative. Mean pairwise distances within the ingroup varied from 0.2–6.9%. There were six synapomorphic indels, with a size ranging from 1–5 bp. All the sequences included in the nrITS matrix were complete except for *Thunia alba*, which lacked 110 characters.

The *mat*K matrix was characterized by a fairly high number of missing data, mostly due to amplification failures. Samples lacking approximately half (800 bp) of the entire *mat*K sequence included *Dendrochilum apoense* T.Hashim., *Dendrochilum septemnervium* H.A.Pedersen and *Dendrochilum tortile* H.A.Pedersen. The *mat*K alignment consisted of 1,783 positions, with a mean ungapped length of approximately 1,769 bp. The alignment contained the least variable sites out of the three alignments, 91 sites (5.1%) with 5 potentially phylogenetically informative sites. Mean pairwise distances within the ingroup ranged from 0.8–71.1%. There was a synapomorphic indel of 1 bp.

The *ycf*1 alignment consisted of 1,297 positions with mean ungapped length of 1065 bp. The number of variable sites was similar to that of nrITS, 244 (18.8%). Out of these positions, 30 were phylogenetically informative. There was one synapomorphic indel with a size of 9 bp. Mean pairwise distances within the ingroup varied from 10–33.5%. Amplification failures for *Dendrochilum septemnervium* resulted in almost half of the desired *ycf*1 marker missing for this species.

**Figure 1.**
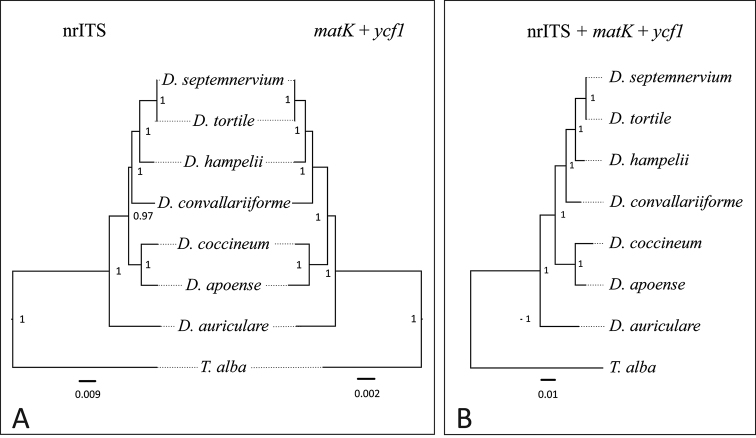
Phylogenetic relationships amongst the sampled species of *Dendrochilum*, created using BEAST and PAUP*. The values on the nodes represent posterior probabilities, whereas branch lengths indicate to the relative number of changes: **A** comparison between topologies based on nrITS and *mat*K + *ycf*1 matrices **B** topology resulting from the combined nrITS + *mat*K + *ycf*1 data matrices.

The phylogenetic trees based on the combined *matK* + *ycf*1 matrix obtained by all three methods yielded overall stronger branch support relative to that of nrITS (Fig. 1A). The internal nodes gained high (PP = 0.97–1.0) support values, and the preceding replicates showed consistent topologies as well as well-supported clades. Clades presented by the *mat*K + *ycf*1 tree were highly congruent with the nrITS tree, and with higher support value.

No hard incongruence was present between the nrITS and the plastid phylogenetic trees obtained by all three methods. The combined nrITS + *mat*K + *ycf*1 matrix yielded a single tree with highly consistent topology and strong support values; the same clades as those in the separate nrITS and *mat*K + *ycf*1 analyses were identified (Fig. 1B). In all our trees, ‘Big Pink’ was positioned as a sister to *Dendrochilum tortile* and *Dendrochilum septemnervium*, which were very closely related, in terms of relative branch length (Fig. 1).

**Figure 2.**
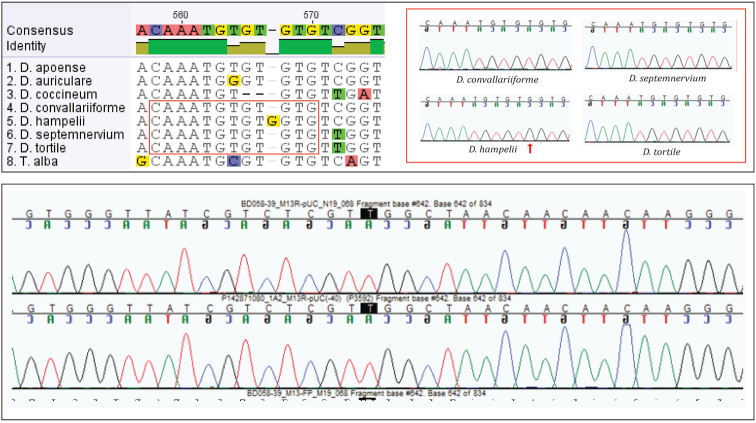
Above: alignment of nrITS sequences of the ingroup species from our phylogenetic analyses. For ‘Big Pink’ (*Dendrochilum hampelii*) and its apparently closest relatives among our study species, electropherograms are shown in red boxes. The electropherograms show clear distinct peaks; the species-specific mutation of ‘Big Pink’ is indicated by a red arrow. Below: electropherogram of ‘Big Pink’ (*Dendrochilum hampelii*) covering a larger region of nrITS; the distinct single peaks in both the forward and reverse sequences suggest this is a wild species rather than an artificial hybrid (see text for details).

Analysis of the nrITS sequence alignment revealed a species-specific mutation of ‘Big Pink’ at position 567 (Fig. 2). Electropherograms of the ITS sequences of ‘Big Pink’ and its three closest relatives among the study species showed clear, distinct signals for positions included in the alignment, including the ‘Big Pink’ species-specific mutation site (Fig. 2).

## Discussion

Nuclear sequence variation found was largely in agreement with previous studies in Coelogyninae (Gravendeel et al. 2001). In general, sequence divergence of nrITS between different species of *Dendrochilum* is known to vary between 0.2–20% (Pedersen et al. 2004; Sulistyo, unpublished data). Additionally, the nrITS sequence obtained from *Dendrochilum hampelii* is not likely to be a paralogous locus since branch lengths are similar to the sequences of other *bona fide* species analyzed. The position of ‘Big Pink’ was in agreement with preliminary analyses based on larger sampling (Sulistyo, unpublished data), as well as with the analyses based on different sequences. We found no evidence suggesting that the morphologically distinctive ‘Big Pink’ could be an artificial hybrid rather than a new wild species. Firstly, the separate phylogenetic analyses of nuclear and plastid markers proved highly congruent. Secondly, the electropherogram of the nrITS sequence of ‘Big Pink’ showed distinct single peaks with no indication of any heterozygosity of the specimen examined.

Genetically, ‘Big Pink’ possessed a number of automorphic mutations compared to the other species included in our small phylogenetic study. These unique mutations were found in nrITS and *ycf*1, whereas one in *mat*K is in need of verification due to the alignment being characterized by a fairly high number of missing data. Morphologically, ‘Big Pink’ is most similar to *Dendrochilum propinquum* Ames. Unfortunately, no material was available of *Dendrochilum propinquum* for DNA sequencing for this study. However, ‘Big Pink’ has much larger flowers (approximately twice the size of those of *Dendrochilum propinquum*), its flowers have petals that are 1.4–1.5 times as broad as the sepals (0.8–1.1 times in *Dendrochilum propinquum*), its labellum is broadly cordate (broadly elliptic to ovate in *Dendrochilum propinquum*), and the stelidia of its column are acute (obtuse in *Dendrochilum propinquum*). Against this background, we describe ‘Big Pink’ as a new species below.

Contrary to the studies of Wanntorp et al. (2002) and Pedersen et al. (2004), the taxon sampling underlying the phylogenetic analyses in this study was too small to allow for any inference of a probable geographic origin of ‘Big Pink’. However, in a preliminary study based on systematically and geographically much broader sampling of *Dendrochilum* s.l., individual phylogenetic analyses using sequences from nrITS, plastid *mat*K, and *ycf*1 have all indicated ‘Big Pink’ to be nested within a clade nearly exclusively consisting of Philippine endemics (Sulistyo, unpublished data). In subsequent field surveys by the second author, a total of 12 plants were observed in February 2014 in the northern provinces of Bukidnon and Misamis Oriental on the island of Mindanao at high elevation, a few of which were flowering. In March 2015, only 4 individuals were observed *in situ* in the wild by the second and third author, and none were flowering. These observations confirm that *Dendrochilum hampelii* is indeed a Philippinese, almost certainly endemic species. It is conjectured to have entered cultivation in Europe through the many domestic markets in Southeast Asia that sell orchid species. For these markets, wild plants are harvested but traded as ‘cultivated’ to circumvent CITES legislation. All Coelogyninae are listed on Appendix 2. Despite this legal protection, illegal trade is continuing at international orchid shows and by web based orders from buyers of specific species or nursery owners hoping to incorporate desirable wild traits into new hybrids (Hinsley et al. 2015).

## Taxonomic treatment

Based on these results, it is determined that “Big Pink” is a new species in need of recognition. Formally naming the species is relevant for horticulture and *ex situ* conservation, because the name provides an unambiguous way to refer to the species.The morphology of ‘Big Pink’ was described using terminology of the vocabulary and list of individual absolute terms in Stearn (1983), if relevant standardized according to the Orchidaceae glossary in Pedersen et al. (2011).

### Dendrochilum hampelii

Taxon classificationPlantaeAsparagalesOrchidaceae

Sulistyo, Gravend., R.Boos & Cootes sp. nov.

urn:lsid:ipni.org:names:77150223-1

Figs 3 , 4

#### Type.

Sine loco et anno, *Perry 490* (holotype L!).

#### Diagnosis.

This new species is similar to *Dendrochilum propinquum* Ames, but is distinguished by its larger flowers with petals proportionally broader (1.4–1.5×) than the sepals, a broadly cordate labellum (6.8–8.0 × 7.2–7.6 m) and acute stelidia.

Medium-sized, tufted epiphytic herb. *Roots* appearing from the rhizome, ca. 2.7 mm in diameter. *Pseudobulbs* tightly clustered on a short rhizome, fusiform, 3.5–5.0 cm long, 0.5–1.4 cm in diameter, longitudinally striated when dry, 1-leaved, initially covered by ca. 3 imperfectly to nearly perfectly tubular, rounded to acute cataphylls that soon disintegrate into persistent fibers. *Leaves* convolute, dorsiventrally flattened, petiolate; petiole channeled, 3.0–4.5 cm long; lamina (ob)lanceolate, obtuse, 13.0–20.0 × 3.7–5 cm, subcoriaceous, with 7–8 distinct (and many indistinct) nerves. *Inflorescence* synanthous, racemose; peduncle suberect, arched, slender, somewhat flattened, 18.0–21.2 cm long, sparsely and finely setose; rachis pendent with distichously alternating flowers (but the rachis axis twisted so as to produce a cylindrical inflorescence), many-flowered with internodes of 3–7 mm, somewhat furrowed, 20.0–27.5 cm long, sparsely and finely setose, basally with 1 appressed non-floriferous bract; flowering starting from the proximal part of the rachis. *Floral bracts* glumaceous, broadly lanceolate to (ovate-)oblong when flattened, obtuse to acute, 4.0–9.5 × 2.2–4.3 mm, entire, 9- to 19-nerved from the base, finely setose on the dorsal side. *Flowers* non-resupinate, pinkish salmon-coloured (Fig. 4A–B) or pale yellow (Fig. 4C) with yellow anther. *Sepals* recurved with revolute margins, entire, obtuse to rounded, minutely mucronate, glabrous, 3- to 5-veined from the base; dorsal sepal lanceolate-oblong, 8.7–11.0 × 3.5–3.7 mm when flattened; lateral sepals ovate-oblong, slightly oblique, 8.1–10.0 × 3.7–4.0 mm when flattened. *Petals* recurved with flat margins, broadly (ovate-)elliptic, often with a subbasal fold in either side, rounded to acute, 8.4–11.1 × 5.4–5.7 mm, 1.4–1.5 times as wide as the sepals, entire, glabrous, 3- to 5-veined from the base. *Labellum* firmly attached, sessile, describing a right to obtuse angle to the column, flat, broadly cordate with entire margins, acute to short-acuminate, 6.8–8.0 × 7.2–7.6 mm, without ornaments, glabrous and smooth, 5- to 7-veined from the base. *Column* suberect, straight, semiterete, 1.7–2.1 mm long, smooth, distally prolonged into a truncate to obscurely 3-lobed wing that distinctly exceeds the anther; stelidia appearing from the distal part of column proper, erect, falcately triangular-oblong, acute, subequal to the apical wing; anther circular to transversely elliptic in upper view, rounded in front, lobed at the back, with a small wart on top; pollinia 4, ellipsoid, devoid of caudicles; rostellum slightly protruding, flat, semicircular; fertile stigma part crescent-shaped, concave. *Ovary* (including pedicel) subterete, slightly longitudinally furrowed, twisted through 180°, distally incurved, 3.8–4.5 mm long, glabrous. *Fruit* not seen.

**Figure 3.**
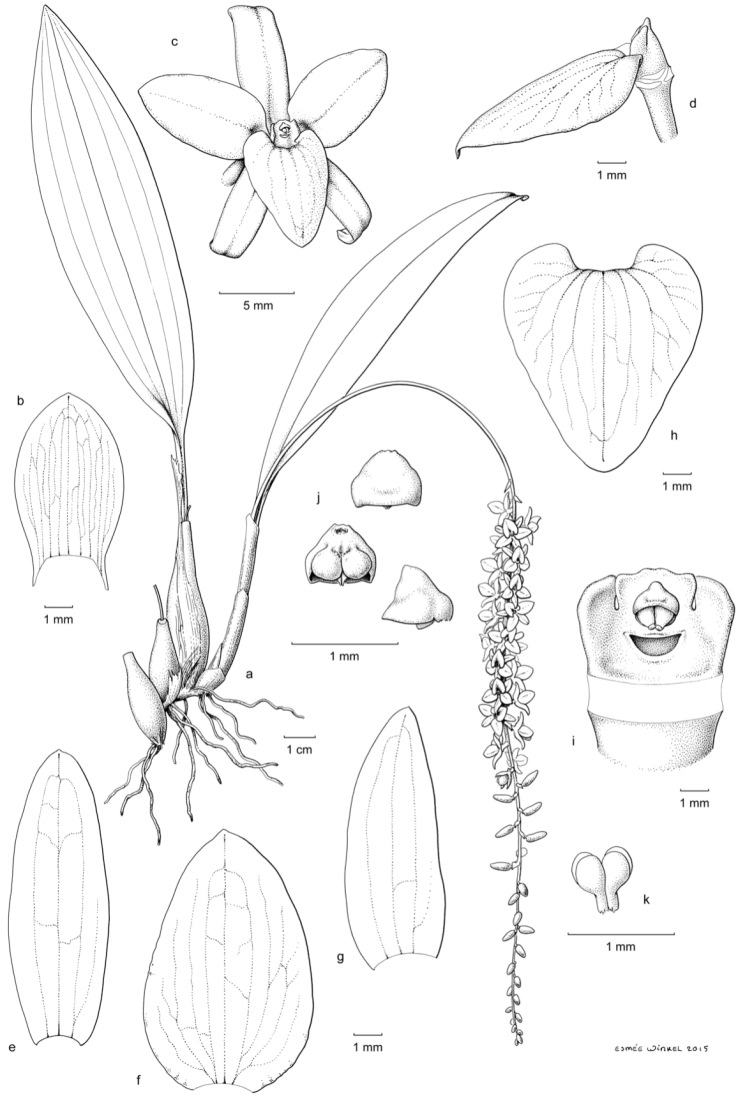
*Dendrochilum hampelii*: **a** habit **b** floral bract **c** flower **d** flower (sepals and petals removed) **e** dorsal sepal **f** petal **g** lateral sepal **h** labellum **i** column, front view **j** anther **k** pollinia. Drawing by Esmee Winkel based on *Hort. bot. Leiden 20130654* (L! [spirit no. WAG0116920]).

**Figure 4.**
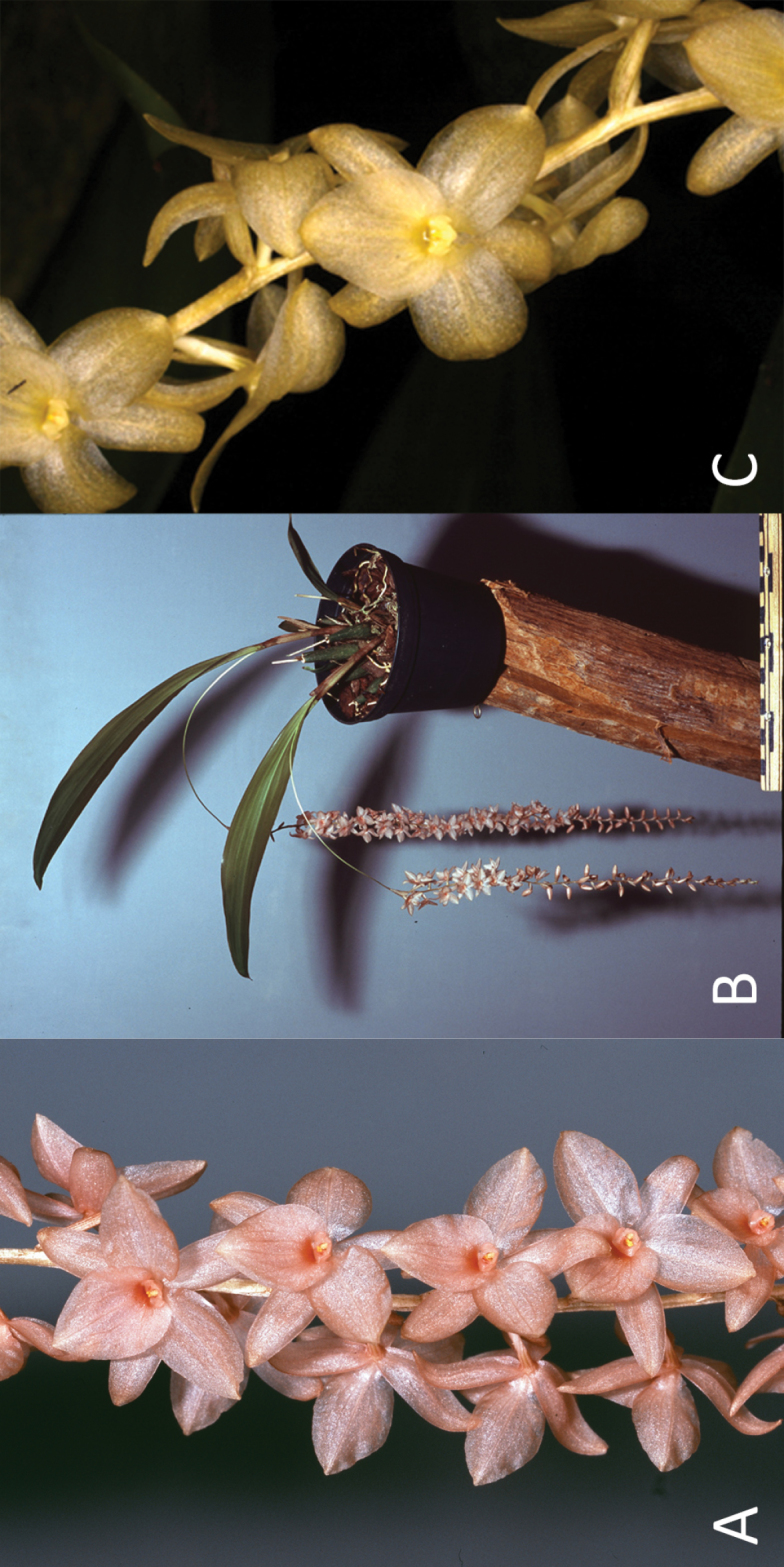
*Dendrochilum hampelii*: **A** portion of inflorescence of cultivated pinkish salmon-coloured form **B** habit. Photographs by Lubbert Westra of *Hort. bot. Leiden 20130654* (L! [spirit no. WAG0116920]) **C** portion of inflorescence of pale yellow-coloured form of a plant growing in the wild in the Philippines in the Misamis Oriental province of the island of Mindanao. Photograph by James Cootes.

#### Additional material examined.

PHILIPPINES? Sine loco et anno, *sine coll./cult. Hort. bot. Leiden 20130654* (L! [spirit no. WAG0116920]).

#### Etymology.

The specific epiphet honours Georg Hampel, who was one of the first to provide us with study material of the newly described species.

#### Distribution and ecology.

The species occurs in the wild in the Philippines in the northern provinces of Bukidnon and Misamis Oriental on the island of Mindanao (Fig. 4C). It grows as an epiphyte at elevations approximately 1,200 m above sea level among mosses on the trunks and branches of trees. Fresh flowers of plants observed in the wild were pale yellow whereas fresh flowers of the cultivated plants studied were pinkish salmon-coloured. We do not consider this reason to describe them as a different variety or forma as color dimorphism is known to occur in other Coelogyninae as well (Gravendeel 2000).

#### Reproductive biology.

The live plant in Leiden flowered in mid-December. Attempts to pollinate flowers of *Dendrochilum hampelii* were made using pollinia from the same flower and pollinia from a different flower in the same inflorescence. None of these efforts led to fruit formation. This indicates that *Dendrochilum hampelii* is probably self-incompatible, as previously demonstrated for *Dendrochilum longibracteatum* Pfitzer (Pedersen 1995), although it should be noted that experimental pollination was severely challenged by the small size of the stigmatic cavity.

#### Conservation status.

Although the species occurs in cultivation we as yet know very little about the distribution and abundance of *Dendrochilum hampelii* in the wild. As such, we recommend the species to be considered for the Data Deficient category of the IUCN Red List of Threatened Species (IUCN 2012).

## Supplementary Material

XML Treatment for Dendrochilum hampelii
